# A random effects variance shift model for detecting and accommodating outliers in meta-analysis

**DOI:** 10.1186/1471-2288-11-19

**Published:** 2011-02-16

**Authors:** Freedom N Gumedze, Dan Jackson

**Affiliations:** 1 Department of Statistical Sciences, University of Cape Town, Rondebosch, 7701, South Africa; 2 MRC Biostatistics Unit, Institute of Public Health, Cambridge, UK

## Abstract

**Background:**

Meta-analysis typically involves combining the estimates from independent studies in order to estimate a parameter of interest across a population of studies. However, outliers often occur even under the random effects model. The presence of such outliers could substantially alter the conclusions in a meta-analysis. This paper proposes a methodology for identifying and, if desired, downweighting studies that do not appear representative of the population they are thought to represent under the random effects model.

**Methods:**

An outlier is taken as an observation (study result) with an inflated random effect variance. We used the likelihood ratio test statistic as an objective measure for determining whether observations have inflated variance and are therefore considered outliers. A parametric bootstrap procedure was used to obtain the sampling distribution of the likelihood ratio test statistics and to account for multiple testing. Our methods were applied to three illustrative and contrasting meta-analytic data sets.

**Results:**

For the three meta-analytic data sets our methods gave robust inferences when the identified outliers were downweighted.

**Conclusions:**

The proposed methodology provides a means to identify and, if desired, downweight outliers in meta-analysis. It does not eliminate them from the analysis however and we consider the proposed approach preferable to simply removing any or all apparently outlying results. We do not however propose that our methods in any way replace or diminish the standard random effects methodology that has proved so useful, rather they are helpful when used in conjunction with the random effects model.

## Background

Meta-analysis typically involves combining summary information from related but independent studies in order to estimate an overall treatment effect. One problem in meta-analysis concerns the presence of outlying studies whose results can excessively influence parameter estimates. If some results appear unusual then it is of course appropriate to check these for errors; in particular if estimates and standard errors of treatment effects have been confused with other quantities then the results from the meta-analysis will be erroneous. We assume here that despite the careful cleaning of data, some study results still appear unusual and are considered potential outliers. In some instances, the identification and understanding of the reason for unusual study results can in itself lead to further understanding of the subject area. With the usual meta-analysis aim of estimating the overall treatment effect in mind, if a small proportion of studies are not in fact truly representative of the population of interest for some unknown reason then their inclusion in the analysis can have unfortunate implications for the resulting inferences. In order to address this type of issue, the Cochrane Collaboration have developed their Risk of Bias tool [1]. This tool enables the identification of studies which have been conducted differently, and in particularly poorly, and provides a means to explain some unusual findings. This type of exercise, which looks for possible reasons for any unusual results, is highly recommended when performing meta-analyses. Related investigations, which might identify unusual patient populations or study practices, are another way to explain the presence of study results which appear strange if taken at face value.

Despite the usefulness of such investigations, it is sometimes the case that some study results are apparent outliers whose cause is unclear. In such instances there is the natural concern that if these study results are included in the analysis but they are not truly representative of the population of interest then misleading inference is almost inevitable. Excluding trial results based on their findings is another source of bias however and the presence of unexplained and unusual study results places the statistician in an uncomfortable situation. Outliers in regression analysis have been widely researched [2,3], but research into outliers in meta-analysis is much more recent [4,5]. If any outliers contribute very little weight to the analysis, because the studies that provide them are small for example, the choice between their inclusion or exclusion is moot; 'outlier' is not synonymous with 'influential'. Large studies are inevitably very influential but need not be outliers, for example. However outlying results are prone to be influential and their presence immediately raises further issues and concerns about any formal statistical inference that might be attempted.

In this paper we propose a random effects variance shift outlier model (RVSOM). This model initially allows the identification of any apparent outliers under the standard random effects model for meta-analysis. This approach is useful because the identification of outliers is in itself problematic. For example, a very large study may initially appear consistent with the rest of the data, but on closer inspection it might provide an estimate much further from the pooled estimate than we expect by chance. Under such circumstances, it may be that this study is an outlier, rather than a smaller study with a more extreme point estimate, which might at first glance be a more obvious candidate.

If any study or studies are identified as outliers, then the RVSOM model further allows their downweighting. The extent of this downweighting depends on how unusual the outliers appear to be, and takes into account the studies' estimated treatment effect and the variance structure. This approach can be used to complement the more usual random effects analysis as a secondary or sensitivity analysis and we consider this approach more satisfactory than performing analyses that simply omit any outliers although these analyses may also be performed if desired. The model underlying the RVSOM was also considered by [6] in the context of outliers in the linear mixed model. Gumedze *et al*. [6] presented a model for accommodating outliers as observations with inflated variance, an outlier being indicated by the size of the associated shift in the variance. The RVSOM differs in that it down-weights observations in the presence of the random effects model for meta-analysis which has the residual variance matrix regarded as known when pooling the studies' results.

The paper is set out as follows. Firstly, we present a random effects variance shift outlier model RVSOM for the random effects model for meta-analytic data. Secondly, we show how this model may be used in practice. In particular, we propose a parametric bootstrap procedure to generate the empirical distribution of the resulting likelihood ratio test statistics and to account for multiple testing using these statistics. The proposed approach is then applied to three meta-analytic data sets, two of which come from the Cochrane Collaboration. Finally, we conclude the paper with a discussion.

## Methods

### A RVSOM in meta-analysis

#### The standard random effects model

We base our modelling on the standard random effects model [7-10] for meta-analysis,

(1) $$y\;=\;\mu 1_{n}+u+e,$$

where ***y ***is a *n*-vector of estimated treatment effects for the *n *independent studies, *μ *is the unknown overall treatment effect, **1***_n _*is a *n*-vector of ones, ***u ***is a *n*-vector of unknown random effects, ***u ***~ *N*(**0**, *τ*^2^**I***_n_*) where *τ*^2 ^is the between-study variance which is unknown. *e *represents residual errors with *e *~ *N*(**0**, ***R***), where $R\;=\;diag(\sigma_{1}^{2},\sigma_{2}^{2},...,\sigma_{n}^{2})$. The elements of ***R***, the study variances, are regarded as known [8], although in reality they are estimated from the *n *independent studies using standard methods. The variance-covariance matrix of (1) can then be written as

(2) $$\mathrm{var}(y)\;=V=\tau^{2}I_{n}+R,$$

with the variance of the *i*th study treatment effect given as $\mathrm{var}(y_{i})=\;\tau^{2}+\sigma_{i}^{2}$.

We will obtain inferences using restricted maximum likelihood estimation (REML). This is a standard approach in the context of meta-analysis [10] and by using a likelihood based approach we can easily fit models with the flexible variance structure we require. We use restricted maximum likelihood rather than the maximum likelihood because we wish to avoid the underestimation of *τ*^2^; any underestimation of the between-study variance might unnecessarily exacerbate the appearance of unusual and hence outlying results. The REML log-likelihood function for model (1) is [10]

(3) $$RL(\tau^{2};y)=-\frac{1}{2}\left\{\sum_{i=1}^{n}\left[\log(\tau^{2}+\sigma_{i}^{2})+\frac{{\left(y_{i}-\hat{\mu }\right)}^{2}}{\tau^{2}+\sigma_{i}^{2}}\right]+\log\sum_{i=1}^{n}{\left(\tau^{2}+\sigma_{i}^{2}\right)}^{-1}\right\},$$

where *RL *denotes the restricted log-likelihood function and

$$\hat{\mu }=\frac{\sum_{i=1}^{n}y_{i}\text{​}{\left(\tau^{2}+\sigma_{i}^{2}\right)}^{-1}}{\sum_{i=1}^{n}{\left(\tau^{2}+\sigma_{i}^{2}\right)}^{-1}}.$$

The associated REML estimate of the between-study variance parameter from this model is denoted ${\hat{\tau }}^{2}$. In general, there is no analytic form for this estimate and it is obtained by using an iterative algorithm which maximises (3) numerically. Several statistical software packages are available for fitting the model (1) such as GenStat [11], R [12], SAS [13] and STATA [14]. Model (1) has the form of a variance components model and can also be fitted with any statistical software that allows for fitting the appropriate linear mixed model with **R **mixed and *τ*^2 ^estimated from the data [10]. If ${\hat{\tau }}^{2}=0$ then the random effects model collapses to a fixed effects model. Upon approximating *τ*^2 ^with ${\hat{\tau }}^{2}$, inferences for the treatment effect are easily obtained in the standard way [9] but this standard procedure requires a reasonably large sample size to perform well in practice [15].

#### Extending the random effects model to the RVSOM

The random effects variance shift outlier model (RVSOM) for the *j*th study (which allows an inflated variance for the *j*th study) takes takes the form

(4) $$y=\mu 1_{n}+\delta_{j}d_{j}+u+e,$$

which adds an extra term *δ_j _**d**_j _*to model (1), where ***d**_j _*is the *j*th unit vector of length *n*, i.e. with value 1 in the *j*th position and zero elsewhere, and *δ_j _*is an unknown random coefficient with $\delta_{j}∼\;N(0,\omega_{j}^{2})$ for $\omega_{j}^{2}\geq 0$. The subscript *j *indicates which study has an inflated variance. Model (4) has the form of a simple linear mixed model with *δ_j _*as a random effect with variance $\omega_{j}^{2}$. The variance-covariance matrix for the data under the RVSOM (4) for the *j*th observation is

$$\text{var(}y\text{)}=\omega_{j}^{2}d_{j}{d^{\prime }}_{j}+V.$$

If we further define $\omega_{i}^{2}=0$ for *i *≠ *j*, the REML log-likelihood function for this model is

$$RL_{\left(j\right)}(\omega_{i}^{2},\tau^{2};y)=-\frac{1}{2}\left\{\sum_{i=1}^{n}\left[\log(\omega_{i}^{2}+\tau^{2}+\sigma_{i}^{2})+\frac{{\left(y_{i}-\hat{\mu }\right)}^{2}}{\omega_{i}^{2}+\tau^{2}+\sigma_{i}^{2}}\right]+\log\sum_{i=1}^{n}{\left(\omega_{i}^{2}+\tau^{2}+\sigma_{i}^{2}\right)}^{-1}\right\},$$

where

$$\hat{\mu }=\frac{\sum_{i=1}^{n}y_{i}{\left(\omega_{i}^{2}+\tau^{2}+\sigma_{i}^{2}\right)}^{-1}}{\sum_{i=1}^{n}{\left(\omega_{i}^{2}+\tau^{2}+\sigma_{i}^{2}\right)}^{-1}},$$

is the estimate of *μ *from model (4). The REML log-likelihood function is now a function of both *τ*^2 ^and $\omega_{j}^{2}$, so there are two parameters to estimate under the RVSOM for the *j*th observation from the restricted log-likelihood. As for the random effects model, once the variance structure has been estimated inference for the treatment effect is straightforward because the study variances are regarded as fixed and known when pooling the studies' results.

The random effects model assumes that the studies' treatment effects are normally distributed and exchangeable. This exchangeability assumption is crucial for the identification of the RVSOM. If instead, for example, fixed effects were used for ***u ***then $w_{j}^{2}$ would not be identifiable. Only one study contributes a term involving $w_{j}^{2}$ to the full likelihood. We can therefore expect $w_{j}^{2}$ to be weakly identified and so rather than use the RVSOM for the primary analysis, we advocate its use to identify outliers and in the context of a sensitivity analysis. We assess whether there is evidence that $w_{j}^{2}$ is greater than zero for a particular study using a parametric bootstrap procedure below, where we produce the replications under the fitted random effects model. If a confidence interval for $w_{j}^{2}$ was required then this could be obtained by modifying this procedure, so that the bootstrap replications were produced using the fitted RVSOM, and obtaining the confidence interval from the resulting distribution's percentiles. The normality assumption for *δ_j _*is made for relative simplicity, because some distributional assumption is needed to make progress and it is a natural assumption to make, but it would be of interest to investigate how sensitive our procedure is to this assumption.

A RVSOM for observation *j *provides an estimate of the shift in the error variance associated with that observation. A large shift may indicate a possible outlier and can be used to down-weight the observation(s) if required. However, an objective measure to indicate this down-weighting needed, which we provide in the next section.

An extension of model (4) which allows different inflated variances for more than one study can be written as

$$y=\mu 1_{n}+D_{I}\delta_{I}+u+e,$$

where ***I ***is a subset {1, 2,..., *r*} of studies considered to be outliers, ***D ***= [***d**_j_*] is an *n × r *matrix containing entires of 0 and 1, where an entry of 1 in the *i*th row and *j*th column indicates that study *i *has the *j*th of *r *inflated variances, and ***δ_I _***is a *r *× 1 vector of unknown random effects. We refer to this model as an 'extended RVSOM'.

### Implementation of the RVSOM

Having fitted the random effects model and made the usual inferences, there may be the concern that outliers are present and that these might have unfortunate implications for the resulting inferences. We then suggest initially fitting the RVSOM to each observation in turn. If a large ${\hat{\omega }}_{j}^{2}$ is obtained when fitting the RVSOM to the *j*th observation then the size of ${\hat{\omega }}_{j}^{2}$ may indicate that this observation is an outlier. We therefore need to determine what constitutes a large $w_{j}^{2}$ in relation to the size of the study and the other variance components.

We consider the use of the likelihood ratio test (LRT) to evaluate the null hypothesis $H_{0}:\omega_{j}^{2}=0$ against the alternative $H_{A(j)}:\omega_{j}^{2}>0$ for a RVSOM for observation *j *(4). The standard LRT statistic provides a means to test this hypothesis and takes the form

(5) $$LRT_{j}=2\left\{RL({\hat{\tau }}^{2};y)-RL_{\left(j\right)}({\hat{\omega }}_{j}^{2},{\hat{\tau }}^{2};y)\right\}.$$

The LRT statistic is analytically intractable except in special cases, but readily obtained from standard mixed model software. Although the restricted log-likelihood is suitable for constructing likelihood ratio tests for variance components provided the mean structure of the null and alternative models are the same [16], the standard asymptotic theory which relates the distribution of LRT statistic to a χ^2 ^distribution under the null does not apply here. This is because the null hypothesis falls on the boundary of the parameter space and regularity conditions are not met [17]. Stram and Lee [18,19] showed that the asymptotic null distribution of the LRT for testing this type of hypothesis is a 50:50 mixture of two chi-squared distributions on zero and one degree of freedom. However, their results assumed either that the data values were independent and identically distributed, or that the data set could be partitioned into a number of independent subsets such that the number of subsets increased with the size of the data set [20]. For a RVSOM model, these conditions cannot be met, as the variance shift applies to a single observation and we encounter all the issues associated with multiple testing. Following Gumedze *et al*. [6], we use a parametric bootstrap procedure to obtain the distributions of the LRT statistics (5).

#### Empirical distribution of the LRT statistic and multiple testing

We propose the following parametric bootstrap procedure to obtain empirical distribution of the likelihood ratio test statistics under the null hypothesis that no outliers are present in the data.

Step 1: Fit the null model defined by (1) to the data to obtain estimates $\hat{\mu }$ and ${\hat{\tau }}^{2}$.

Step 2a: Generate a new data vector

$$y^{*}=\hat{\mu }1_{n}+u^{*}+e^{*},$$

where u* is randomly generated as$N(0,{\hat{\tau }}^{2}I_{n})$, and e* is randomly generated as *N*(**0**, ***R***). Fit the null model to y*.

Step 2b: Compute the likelihood ratio test statistics *LRT_j_, j *= 1,..., *n*, by fitting the RVSOM (4) to the simulated data y* for each observation in turn and compute and save the order statistics of the set {*LRT_j_*; *j *= 1... *n*}.

Step 3: Repeat steps 2a and 2b *R *times, for *R *reasonably large, for example *R *= 5000. This step generates the bootstrap replications and hence an empirical distribution of size *R *for each order statistic.

Step 4: Calculate the 100(1-*α*)th percentile for each order statistic for the required significance level *α*. The percentiles using *α *= 0.05 and for the *k *= 1 (largest LRT statistic), *k *= 2 (second largest LRT statistic) and *k *= 3 order statistics are shown in the plots given in next section. These percentiles provide thresholds for assessing whether up to three studies are outliers under the random effects model.

#### Identifying and downweighting outliers

Using any significance level alpha for the LRT order statistics, the presence of outliers can be formally assessed by placing the LRT statistics in descending order and regarding any set of *r *studies that provide the largest LRT statistics and reach the corresponding threshold are regarded as outliers. Since our method has identified these studies' findings as being unusual, it is pertinent to check the conduct and protocols of these trials carefully to see if any explanation can be found for their apparently outlying results.

If there is no apparent reason to exclude these trials, perhaps from the Cochrane Risk of Bias tool for example, then the concern that they might be unduly influential but excludable on some unforseen grounds persists. In such circumstances a simple approach is to consider sensitivity analyses where some or all of these trials are excluded but simply discarding entire trials is rather extreme and various combinations of exclusions might be contemplated in this procedure. Instead of this deletion of observations we propose fitting an 'extended RVSOM' where separate and additional variance components $\omega_{j}^{2}\geq 0$ are allowed for all of the outlying trials. Since these additional variances cannot be negative, the extra variability permitted by this model downweights the apparent outliers, where this downweighting depends on how unusual their results appear to be. If the study results are truly disparate from the rest, the additional variance components will be enormous and the corresponding studies will have negligible weight but more commonly the effect will be to downweight the apparent outliers.

## Results and Discussion

In this section we analyze three meta-analytic data sets, two of which come from the Cochrane Collaboration. The two data sets: CDP-choline for cognitive and behavioural disturbances, and Fluoride toothpaste for preventing dental caries have been previously analysed [4]. These provide contrasting examples. We will use the conventional *α *= 0.05 in our procedure for identifying outliers described as above but this could be altered to reflect a priori beliefs about the possibility and impact of the presence of outliers.

### CDP-choline for cognitive and behavioural disturbances

**Figure 1:**
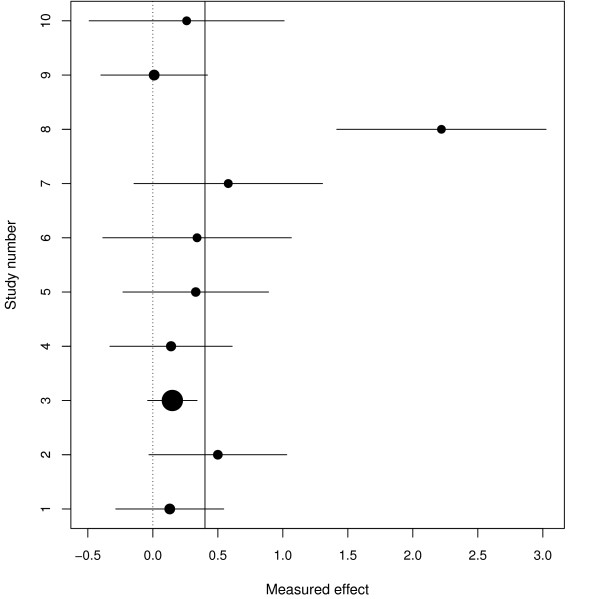
**Forest plot for the CDP study**. Solid vertical line represents the value of the treatment effect under a random effects model. The centre of each circle in the confidence interval for each study represents the treatment effect from that study. The size of each circle is inversely proportional to the total variance under a random effects model.

Fioravanti and Yanagi [21] present a meta-analysis of cytidinediphosphocholine (CDP-choline) for cognitive and behavioural disturbances associated with chronic cerebral disorders in the elderly. There are ten studies for the outcome used here, memory measures, and the *y_i _*are standardised mean differences of treatment versus placebo with positive values of *y_i _*indicating that the treatment is beneficial. The forest plot shows the 8th study as an apparently obvious outlier and none of the other results appear unusual (Figure 1). This example provides a relatively straightforward test of our methods.

**Figure 2:**
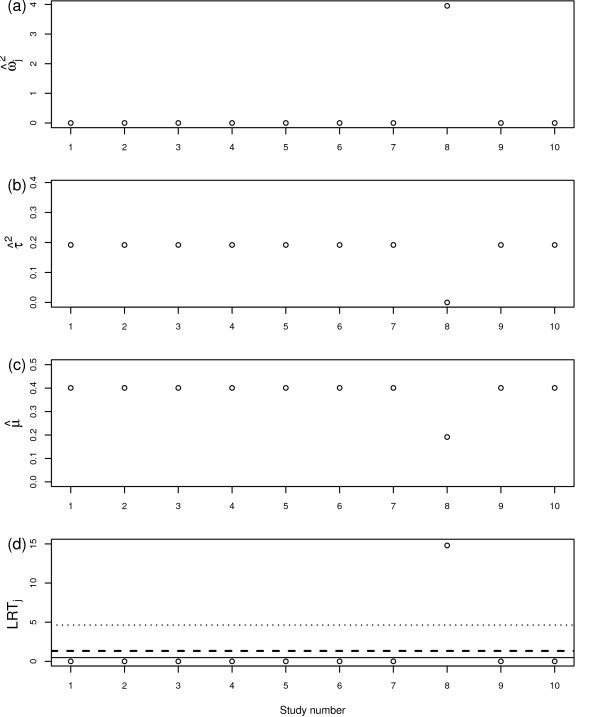
**RVSOM statistics plotted against study number for the CDP study**. (a) Variance shift estimates, ${\hat{\omega }}_{j}^{2}$. (b) Treatment effect estimates $\hat{\mu }$. (c) Random effect variance estimates ${\hat{\tau }}^{2}$. (d) Likelihood ratio test statistics for a RVSOM for study *j*, plotted against study number. 95th percentile of the empirical distribution under the null hypothesis shown for the first *k *order statistics for each test: *k *= 1 (dotted line), *k *= 2 (dashed line) and *k *= 3 (solid line).

Figure 2 and Table 1 show the results for the RVSOM analyses for the CDP-choline data set. The first plot (a) in Figure 2 shows the estimated $\omega_{j}^{2}$ from the *j*th RVSOM and the next two plots (b-c) show the corresponding estimates of the between-study variance and the treatment effect. The final plot (d) shows the likelihood ratio test statistics from which we see that observation 8 is clearly detected as an outlier as expected; in particular its LRT statistic is around three times the threshold for the first order statistic. Table 1 shows the inferences for the random effects model (*M*_0_) and the extended RVSOM (*M*_1_; in this case, this is the same as the RVSOM model for the 8th observation because this was the only observation that was designated as a potential outlier). We see that upon allowing for the overdispersion of the 8th trial result the estimate of the between-study variance drops to zero. The outlier is clearly highly influential but its downweighting, which is so huge as to be almost, but not quite, equivalent to removing it, does not threaten the null hypothesis. The estimate of treatment effect is approximately halved however. Model *M*_1 _provides a suitable sensitivity analysis when used in conjunction with the standard random effects model.

**Table 1:** Estimated parameters for models fitted to the CDP data: overall treatment effect (*μ*), variance shift estimates for study *j *($\omega_{j}^{2}$) and between-study variance (*τ*^*2*^). M_0_: Random effects model and M_1_: Extended RVSOM for study 8.

	Model *M*_0_		Model *M*_1_	
Parameter	Estimate	95% CI	Estimate	95% CI
μ	0.401	(0.08;0.72)	0.191	(0.058;0.324)
*τ*^2^	0.192	-	0:000	-
$\omega_{8}^{2}$	-	-	3.951	-

#### Intravenous magnesium in acute myocardial infarction

**Figure 3:**
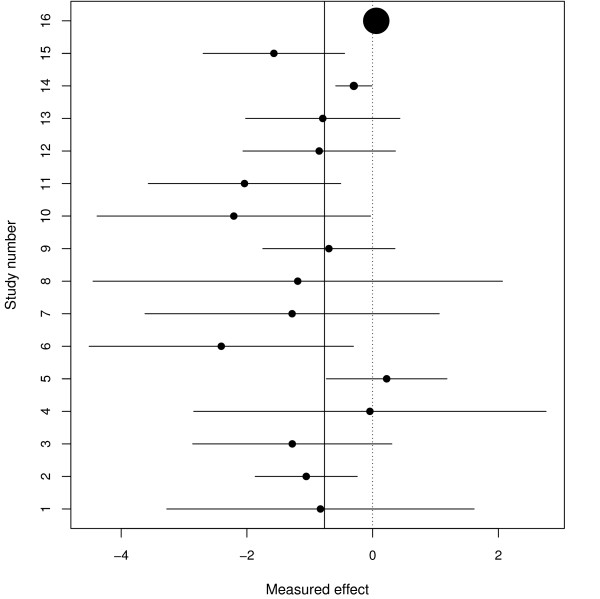
**Forest plot for the magnesium study**. The centre of each circle in the confidence interval for each study represents the treatment effect from that study. The size of each circle is inversely proportional to the total variance under a random effects model.

These 16 trials are a well-known example where the results of a meta-analysis were contradicted by a single large trial [22] (trial 16). The forest plot in Figure 3 does not suggest that any of the studies are outliers but study 16 is at best curious when compared to the other studies and so this example presents a rather unusual challenge to our methods.

**Figure 4:**
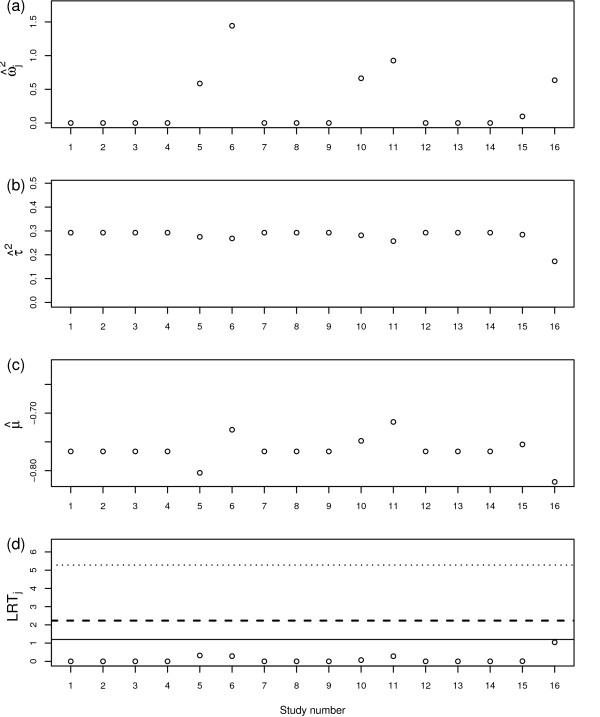
**RVSOM statistics plotted against study number for the magnesium study**. (a) Variance shift estimates, ${\hat{\omega }}_{j}^{2}$. (b) Treatment effect estimates $\hat{\mu }$. (c) Random effect variance estimates ${\hat{\tau }}^{2}$. (d) Likelihood ratio test statistics for a RVSOM for study *j*, plotted against study number. 95th percentile of the empirical distribution under the null hypothesis shown for the first *k *order statistics for each test: *k *= 1 (dotted line), *k *= 2 (dashed line) and *k *= 3 (solid line).

Figure 4 show the results for the RVSOM analyses for the intravenous magnesium in acute myocardial infarction data set. Study 16 is clearly the most influential and although it does not provide the largest $\omega_{j}^{2}$ it does provide the largest likelihood ratio test and we conclude that, although there is very weak evidence for any outliers in these data, it is indeed study 16, rather than study 6 say, which provides the strongest evidence for being an outlier. From a careful inspection of the forest plot, study 16 appears curious in relation to the other studies. Our methodology has performed well, in that it provided results which are in agreement with intuition, by highlighting study 16 but not actually designating it an outlier. The RVSOM for observation 16 provides inferences that are in broad agreement with the usual random effects analysis.

#### Fluoride toothpaste for preventing dental caries

**Figure 5:**
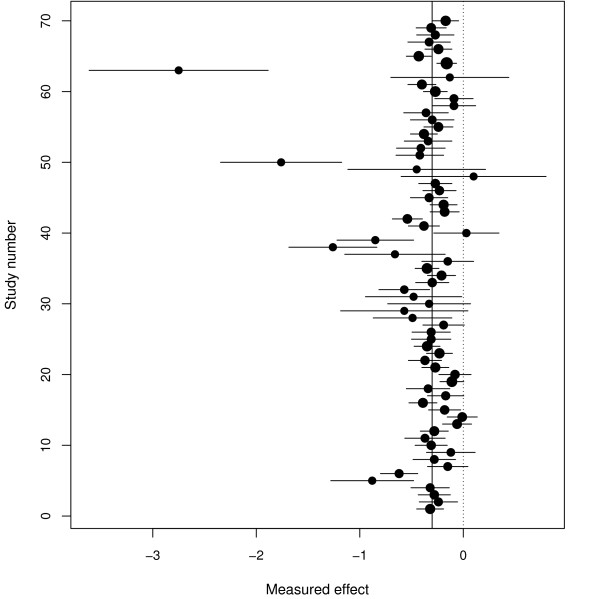
**Forest plot for the flouride toothpaste study**. The centre of each circle in the confidence interval for each study represents the treatment effect from that study. The size of each circle is inversely proportional to the total variance under a random effects model.

Marinho *et al*. [23] present a meta-analysis of fluoride toothpastes for preventing dental caries in children and adolescents. There are 70 studies, and the outcome is the difference between treatment and control of tooth areas with caries; negative values of *y *indicate that the treatment is beneficial. This is a large meta-analysis with obvious outliers, but where the treatment benefit is not in doubt, and there was no suggestion of publication bias in the original review. The forest plot shows studies 50 and 63 as apparent outliers (Figure 5) but there are other possibilities as well, such as study 38, so these data presents a very considerable challenge to our methods compared to the previous examples.

**Figure 6:**
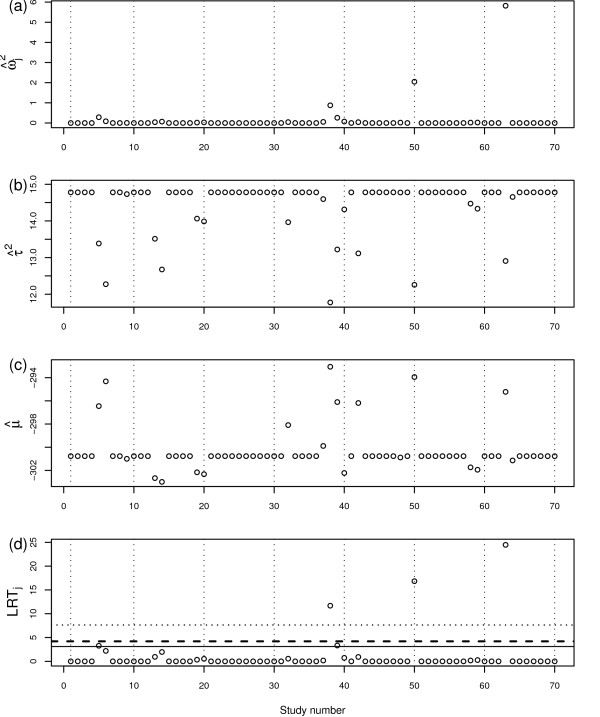
**RVSOM statistics plotted against study number for the flouride toothpaste study**. (a) Variance shift estimates, ${\hat{\omega }}_{j}^{2}$. (b) Treatment effect estimates $\hat{\mu }$. (c) Random effect variance estimates ${\hat{\tau }}^{2}$. (d) Likelihood ratio test statistics for a RVSOM for study *j*, plotted against study number. 95th percentile of the empirical distribution under the null hypothesis shown for the first *k *order statistics for each test: *k *= 1 (dotted line), *k *= 2 (dashed line) and *k *= 3 (solid line).

Figure 6 and Table 2 show the results for the RVSOM analyses for the flouride toothpaste data set. Studies 50 and 63 are clearly (and study 38, but to a lesser extent) identified as outliers but other outlying results appear to be more easily explainable by the random effects model and do not need any additional variation to adequately describe them. Table 2 shows that the inferences are very robust when these three outliers are downweighted using the extended RVSOM (which includes three $w_{j}^{2}$, *j *= 38, 50, 63 terms in a single model) and our findings greatly alleviate any concerns about the potential impact of outliers in these data. Once again, our proposed methodology has performed well.

**Table 2:** Estimated parameters for models fitted to the for the flouride toothpaste data: overall treatment effect (*μ*), variance shift estimates for study *j *(*$\omega_{j}^{2}$*) and between-study variance (τ*^2^*). M_0_: Random effects model and M_1_: Extended RVSOM for studies 38, 50, and 63.

	Model *M*_0_		Model *M*_1_	
**Parameter**	**Estimate**	95% CI	Estimate	95% CI
μ	-0.3008	(-0.33;-0.27)	-0.284	(-0.32;-0.25)
*τ*^2^	0.015	-	0.009	-
$\omega_{38}^{2}$	-	-	0.897	-
$\omega_{50}^{2}$	-	-	2.082	-
$\omega_{63}^{2}$	-	-	5.879	-

## Conclusions

The proposed RVSOM provides a means to identify and, if desired, downweight outliers in meta-analysis. It does not eliminate them from the analysis however and we consider the proposed approach preferable to simply removing any or all apparently outlying results. We do not however propose that our methods in any way replace or diminish the standard random effects methodology that has proved so useful, rather they are helpful when used in conjunction with the random effects model. We note that statistical inferences based on modelling choices that were determined by the outcome of statistical tests, such as ours, are open to question and critique and partly for this reason we present our methods only in the context of sensitivity or secondary analyses. Our methods cannot provide reasons for any apparent outliers but are useful when some findings seem unaccountably unusual and their presence is a cause for concern. We have focused our attention on the notion of outlying trial results, rather than those that are influential, or have high leverage, and so on, but outliers very often exert alarming amounts of influence and these concepts are related. For the three examples considered here, the apparent outliers only had serious implications for the CPD-choline analysis, i.e. different estimated treatment effects result when outliers are downweighted, and hence our methods can either confirm or diminish any fears that inferences are driven by a handful of unusual results.

The likelihood ratio test gives an objective measure for detecting outliers in meta-analysis. Some may consider this objective measure in itself useful and use this part of the methodology alone rather than take the next step and downweight any apparent outliers. Determining which studies might be designated as outliers may be difficult from the visual inspection of plots and our methods could be used to inform this common but usually informal process. The methodology could be applied to aid the identification of any unusual findings and shortlist trials whose protocols and conduct should be examined especially carefully before being entered into the analysis.

We suggest that the results from the extended RVSOM, with inflated variances for any studies considered to be outliers, provides a useful sensitivity analysis. If the resulting inferences for the treatment effect are very different to those from the random effects model then all inferences should be very cautiously interpreted.

The fixed effects version of our model (with *τ*^2 ^= 0) is computationally and conceptually simpler and might be applied under the strong assumption of homogeneity for the non-outlying studies. A concern here however that the almost inevitable between-study variation will result in more variation than the model anticipates and therefore the identification of 'outliers' that are not truly unusual but rather they are due to the usual random variation that occurs between trials. We align ourselves with those who prefer random effects methods for meta-analysis but appreciate the arguments made by those whose opinions differ to ours. We have built our model on the standard random effects model which assumes within and between-study normality. If some studies are small and their within-study normal approximations are not accurate then some apparent outliers may be due to this assumption and this possibility should be considered. Non-normal between-study distributions have also been used in meta-analysis [4,24]. It is interesting that our extended RVSOM produced very similar results for our first and third examples as the heavy tailed distributions for the random effect adopted by Baker and Jackson [4]. Both the extended RVSOM and these heavy tailed random effects serve to down-weight the outliers so this might be expected to be the case more generally. However the methods proposed here avoid integrating out unusual random effects and MCMC methods [24] and retain all the advantages of working with data that are assumed to be normally distributed.

The RVSOM model is not especially appropriate if there are many apparent outliers or the collection of trial results are truly unusual; it is not helpful or meaningful to designate a large proportion of the studies as outliers. In such instances many would balk at the possibility of meta-analysis altogether but heavy tailed or less usual models for the random effect may be useful in such instances, as shown by Baker and Jackson [4].

Our procedure for the computation of thresholds for the likelihood ratio test statistics makes our proposal quite computationally intensive but in the current climate this presents little difficulty. More computationally intensive methods involving bootstrapping and permutation tests are becoming more common proposals in meta-analysis however and we anticipate that this trend will continue.

## Competing interests

The authors declare that they have no competing interests.

## Authors' contributions

Both FNG and DJ conceived the study, performed the statistical analysis and wrote the manuscript. Both authors read and approved the final manuscript.

## Appendix: Computations

The analysis for the three examples given in the manuscript were conducted using Genstat [11] but full R [12] code is available at http://www.mrc-bsu.cam.ac.uk/Software/download.html#RPackages

## Pre-publication history

The pre-publication history for this paper can be accessed here:

http://www.biomedcentral.com/1471-2288/11/19/prepub
